# NOP53 Suppresses Autophagy through *ZKSCAN3*-Dependent and -Independent Pathways

**DOI:** 10.3390/ijms22179318

**Published:** 2021-08-27

**Authors:** Young-Eun Cho, Yong-Jun Kim, Sun Lee, Jae-Hoon Park

**Affiliations:** Department of Pathology, College of Medicine, Kyung Hee University, Seoul 02453, Korea; yefbs@khu.ac.kr (Y.-E.C.); yjkim1@khu.ac.kr (Y.-J.K.)

**Keywords:** autophagy, NOP53, *ZKSCAN3*, nucleolus, LC3B

## Abstract

Autophagy is an evolutionally conserved process that recycles aged or damaged intracellular components through a lysosome-dependent pathway. Although this multistep process is propagated in the cytoplasm by the orchestrated activity of the mTOR complex, phosphatidylinositol 3-kinase, and a set of autophagy-related proteins (ATGs), recent investigations have suggested that autophagy is tightly regulated by nuclear events. Thus, it is conceivable that the nucleolus, as a stress-sensing and -responding intranuclear organelle, plays a role in autophagy regulation, but much is unknown concerning the nucleolar controls in autophagy. In this report, we show a novel nucleolar–cytoplasmic axis that regulates the cytoplasmic autophagy process: nucleolar protein NOP53 regulates the autophagic flux through two divergent pathways, the *ZKSCAN3*-dependent and -independent pathways. In the *ZKSCAN3*-dependent pathway, NOP53 transcriptionally activates a master autophagy suppressor *ZKSCAN3*, thereby inhibiting MAP1LC3B/LC3B induction and autophagy propagation. In the *ZKSCAN3*-independent pathway, NOP53 physically interacts with histone H3 to dephosphorylate S10 of H3, which, in turn, transcriptionally downregulates the *ATG7* and *ATG12* expressions. Our results identify nucleolar protein NOP53 as an upstream regulator of the autophagy process.

## 1. Introduction

Autophagy is an evolutionally conserved cellular process involved in the degradation of abnormal protein aggregates and the removal of damaged cytoplasmic organelles through a lysosome-dependent pathway [1]. It is a set of cytoplasmic processes, including the sequestration of cytoplasmic contents by the phagophores, maturation of autophagosomes, formation of autolysosomes, and ultimate cargo degradation [2]. These cytoplasmic autophagic events are controlled and propagated by the mTOR complex (mTORC) [3], phosphatidylinositol 3-kinase complex (PI3K) [4], and a series of cytoplasmic autophagy-related proteins (ATGs) [5]. However, recent investigations have demonstrated that the nucleus is a major regulator of autophagy [6]. The nuclear translocation of transcription factors such as Gln3 [7], TFEB [8], and FOXO [9] regulates the expression of autophagy-related genes and enhances autophagy. In addition, identification of the microRNAs associated with the autophagic process indicates that nuclear events are key mechanisms for autophagy regulation [10,11,12,13]. Several microRNAs, including miR-17 and miR-30a, are involved in the regulation of ATG genes such as *ATG7*, *ATG2*, *ATG4*, and *ATG16* [11,12]. For example, miR-17 had the effect of inhibiting autophagy by reducing the *ATG7* expression [12]. *Beclin 1* may be inhibited by miR-30a, thereby modulating autophagy activity [13]. Nevertheless, the networks controlling autophagy that encompass the nucleus and cytoplasm remain to be determined.

The nucleolus is a highly dynamic intranuclear structure that senses and responds to signals in important cellular activities, such as cell cycle progression, ribosome biogenesis and metabolism, and apoptosis [14]. Moreover, several recent reports have indicated that the nucleolar protein nucleophosmin (NPM) [15] and alternative reading frame (p19ARF) [16,17] participate in controlling the autophagic process. These observations indicate that the nucleolus is actively involved in the regulation of autophagy flux. However, the signaling pathways through which the nucleolus regulates cytoplasmic events are largely unknown.

Ribosome biogenesis factor NOP53 (NOP53/Pict-1/GLTSCR2) is a nucleolar protein that participates in cell cycle progression, apoptosis, and tumor development [18,19]. Specifically, NOP53 is redistributed to the nucleoplasm under cellular stresses such as hypoxia and DNA damage, where it regulates the stability of the nuclear proteins involved in the stress-responsive process or tumor suppression, including p53 [20,21], NPM [22], and p14ARF [23], thereby allowing cells to adapt to intrinsic or extrinsic stimuli. Interestingly, these stress-responsive proteins regulated by NOP53 play a critical role in autophagy. Thus, it is conceivable that NOP53 may function in autophagy regulation through a p53- or NPM-dependent pathway.

The aim of this study was to investigate the roles of NOP53 in autophagy regulation. In this report, we show that NOP53 regulates cytoplasmic autophagic processes through both *ZKSCAN3*-dependent and -independent pathways. Our results identify NOP53 as a central member of the nucleolar–nuclear–cytoplasmic axis in autophagy regulation.

## 2. Results

### 2.1. NOP53 Suppresses Autophagy

**Figure 1:**
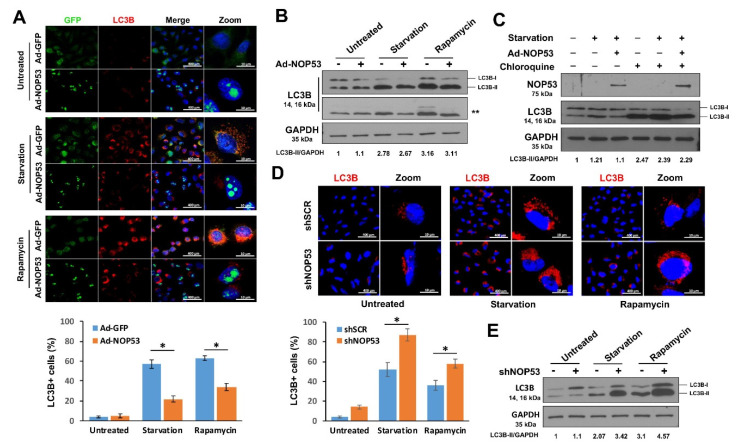
NOP53 suppresses the autophagic flux. (**A**) LN18 cells were untreated or transduced with Ad-GFP or Ad-NOP53/GFP, followed by starvation in EBSS medium for 24 h or treatment with 10 ng/mL rapamycin for 12 h, and immunocytochemical staining with anti-LC3B antibody was performed (upper three panels). Cells with formations of more than 5 LC3B spots were counted, and the ratio is presented (lower panel). Data from three independent experiments are shown as the means ± SD; * *p* < 0.01. (**B**) Cells lysates treated as in “A” were subjected to Western blotting using the anti-LC3B antibody after normalization to *GAPDH*. **, short exposure. (**C**) Cells were treated as indicated in the presence or absence of chloroquine (50 μM) for 3 h, and the lysates were subjected to western blot using anti-NOP53 or anti-LC3B antibody after normalization to *GAPDH*. (**D**) shSCR and shNOP53 cells were untreated, starved in EBSS medium, or treated with rapamycin, as in “A”, and immunocytochemical staining was carried out using the anti-LC3B antibody (upper panels). The cells with LC3B spot formation were counted, and the ratio is presented (lower panel). Data from three independent experiments are shown as the means ± SD; * *p* < 0.01. (**E**) shNOP53 cells were untreated, starved, or treated with rapamycin, and then, Western blotting was performed using the indicated antibody. LC3B-II or *GAPDH* expression using Western blotting was quantified using ImageJ software. The LC3B-II/*GAPDH* ratio was normalized to the control.

NOP53 shifts between the nucleolus and nucleoplasm, where it regulates the stability of the nuclear proteins participating in the stress response or autophagy regulation, such as p53 [21] and nucleophosmin (NPM) [22]. Hence, we investigated whether NOP53 is directly associated with the autophagic process. LN18 glioblastoma cells transduced by adenovirus expressing GFP-tagged NOP53 (Ad-NOP53) or control GFP (Ad-GFP) were starved in Earle’s Balanced Salt Solution (EBSS) medium or treated with rapamycin, and the number of MAP1LC3B (LC3B)-positive cells were counted as an indicator of autophagosomes. The ectopic expression of NOP53 significantly suppressed LC3B spot formation induced by starvation or rapamycin treatment compared to GFP-overexpressing cells (Figure 1A). Consistent with these observations, the conversion of LC3B-I to LC3B-II decreased with the expression of ectopic NOP53 (Figure 1B). Although these findings indicate that autophagosomes with LC3B-II were decreased by NOP53 overexpression, they do not distinguish decreased autophagic flux from enhanced autophagosomal maturation. Thus, we performed a LC3B turnover assay to determine whether NOP53 reduced the autophagic flux. Cells transduced with Ad-NOP53 or Ad-GFP were starved in the presence or absence of the lysosomal inhibitor chloloquine (50 μM) for 3 h, and the cell lysates were subjected to Western blotting. As shown in Figure 1C, NOP53 expression reduced the autophagic flux. Next, we determined whether the downregulation of NOP53 expression effected the autophagic processes. NOP53 knockdown LN18 cells (shNOP53), by a stable transduction with lentivirus-delivering shRNA targeted in NOP53, showed increased autophagy compared to control cells with scrambled shRNA (shSCR) transduction (Figure 1D,E). Taken together, our findings demonstrate that the NOP53 expression level is closely related to the autophagic process.

### 2.2. Suppression of Autophagy by NOP53 Is Independent of p53 or NPM

**Figure 2:**
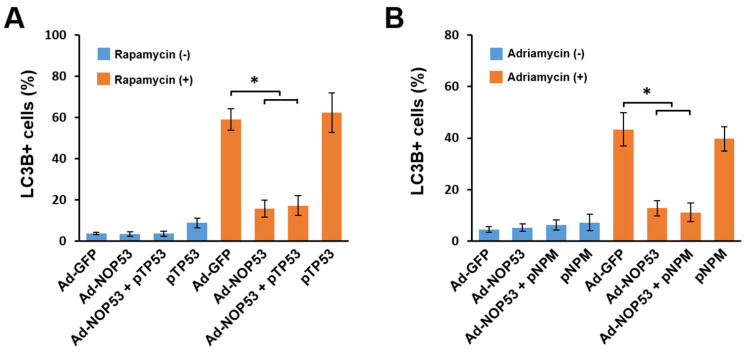
NOP53 suppresses autophagy through the p53- and NPM-independent pathways. (**A**) Cells were singly transduced with a p53−expressing plasmid (p53) or Ad-NOP53 or co-transduced with Ad-NOP53 and p53 in the presence or absence of 10 ng/mL rapamycin, followed by immunocytochemical staining with an anti-LC3B antibody. Cells with LC3B-positive spot formations were counted, and the ratio is presented. Data from three independent experiments are shown as the means ± SD; * *p* < 0.01. (**B**) Cells were singly transduced with NPM−expressing plasmid (pNPM) or Ad-NOP53 or cotransduced with Ad-NOP53 and pNPM in the presence or absence of 10 ng/mL Adriamycin, followed by immunocytochemical staining with an anti-LC3B antibody. Cells with LC3B-positive spot formations were counted, and the ratio is presented. Data from three independent experiments are shown as the means ± SD; * *p* < 0.01.

Previously, we and others showed that NOP53 regulates the stability of p53 [20,21] and NPM [22], which are capable of stimulating autophagy. Thus, we assessed whether autophagy suppression by NOP53 resulted from the enhanced degradation of p53 or NPM. We co-transduced LN18 cells with Ad-NOP53 and p53-expressing plasmids (p53) for 48 h to counteract the enhanced degradation of p53 by NOP53, followed by a rapamycin treatment for an additional 24 h. As shown in Figure 2A, NOP53 suppressed the autophagy induced by rapamycin irrespective of the p53 expression level, indicating that NOP53 suppresses autophagy through a p53-independent pathway. A recent report showed that NPM is essential for the autophagy induced by the Pol I transcription inhibitor [15]. Thus, we carried out similar experiments using the Pol I transcription inhibitor Adriamycin. The ectopic expression of NPM did not affect the NOP53-induced autophagic suppression (Figure 2B). These findings indicate that the autophagy suppression by NOP53 did not result from the enhanced degradation of NOP53-interacting nucleolar proteins.

### 2.3. NOP53 Transcriptionally Upregulates the Expression of Autophagy Repressor ZKSCAN3

**Figure 3:**
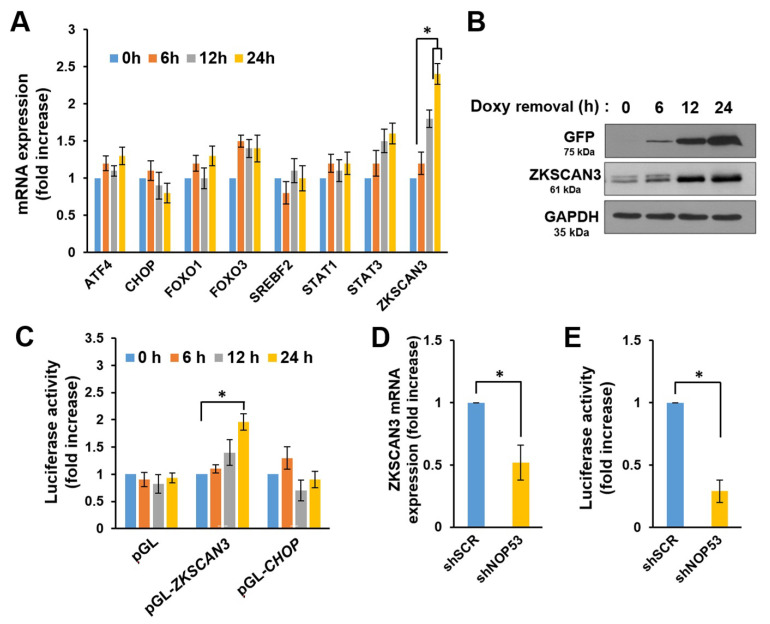
NOP53 transcriptionally regulates *ZKSCAN3*. (**A**) LN18 cells were transduced with doxycycline-inducible (Tet/OFF system) Ad-NOP53 in the presence of 20-ng/mL doxycycline for 24 h. Then, doxycycline was removed at the zero time point, and the cells were harvested after 6, 12, and 24 h. Real-time qPCR was performed as described in “Methods”. Data obtained from three independent experiments represent a fold increase in the mRNA level of each transcriptional factor relative to the basal transcription level. The amount of target mRNA was normalized by determining the level of *GAPDH* mRNA. * *p* < 0.01. (**B**) The cells were treated as indicated in “A”, and the lysates were subjected to Western blots using anti-GFP (ectopic NOP53) or anti-*ZKSCAN3* antibodies after normalization to *GAPDH*. (**C**) LN18 cells transduced by Ad-NOP53 in the presence of 20-ng/mL doxycycline were transfected with mock (pGL) or *ZKSCAN3* promoter (pGL-*ZKSCAN3*) plasmid for 24 h. Then, a luciferase assay was performed at the indicated times after the removal of doxycycline. The histogram represents the fold increase relative to the luciferase activity at the zero time point. Data were obtained from three independent experiments and shown as the means ± SD; * *p* < 0.01. (**D**,**E**) Real-time qPCR of the *ZKSCAN3* mRNA expression (**D**) and a *ZKSCAN3* promoter assay (**E**) on the shSCR and shNOP53 cells were performed. Data were obtained from three independent experiments and shown as the means ± SD; * *p* < 0.01.

To elucidate the mechanisms for autophagy suppression by NOP53, we next considered the possibility that NOP53 regulates the expression level of the transcriptional factors involved in the autophagic process because of its exclusive localization within the nucleus. Hence, we determined the changes in the expression levels of the transcriptional factors known to regulate autophagy, including *ATF4*, *CHOP*, *FOXO1*, *FOXO3*, SREBP2, *STAT1*, *STAT3*, and *ZKSCAN3* [6]. LN18 cells transduced by doxycycline-inducible (Tet/OFF system) Ad-NOP53 were cultured in a medium containing doxycycline, and the cells were harvested after the removal of doxycycline at the indicated times. Real-time qPCR and Western blotting showed a significant upregulation of *ZKSCAN3* by ectopic NOP53 expression both in the mRNA and protein levels (Figure 3A,B), while the other transcriptional factors involved in autophagy were not altered significantly (Figure 3A and data not shown). *ZKSCAN3* is a master transcriptional suppressor of autophagy [24]. To ascertain the transcription-dependent upregulation of *ZKSCAN3* by NOP53, we performed the *ZKSCAN3* and *CHOP* promoter assays in the same set of LN18 cells used for the qPCR and found that NOP53 activated the *ZKSCAN3* promoter. In contrast, the promoter of *CHOP*, a negative control, did not respond to the NOP53 expression (Figure 3C). Next, we carried out a qPCR and a promoter assay on the shNOP53 cells. Knockdown of the NOP53 expression resulted in the suppression of *ZKSCAN3* mRNA expression (Figure 3D) and a decrease of *ZKSCAN3* promoter activity (Figure 3E). To elucidate the mechanism of NOP53-dependent activation of the *ZKSCAN3* promoter, we determined whether NOP53 is recruited to the *ZKSCAN3* promoter using a ChIP assay. However, the direct binding of NOP53 to the *ZKSCAN3* promoter was not noted (data not shown). Taken together, our results indicate that NOP53 transcriptionally regulates the expression of *ZKSCAN3* by activation of the *ZKSCAN3* promoter.

### 2.4. NOP53 Suppresses Autophagy by Downregulating Autophagy-Related (ATG) Genes and Proteins Both through ZKSCAN3-Dependent and -Independent Pathways

**Figure 4:**
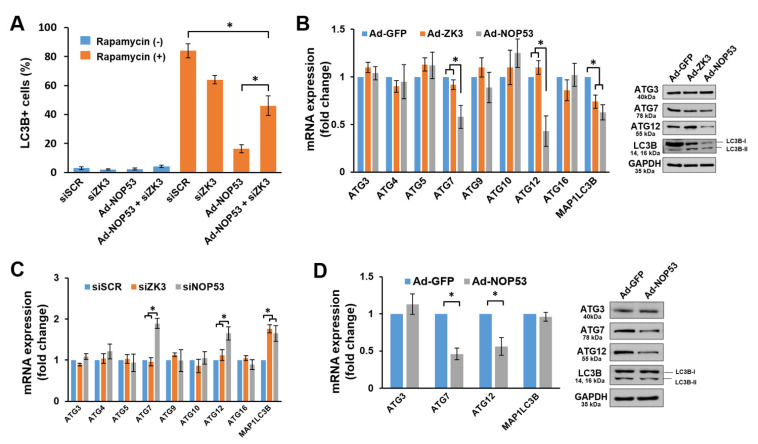
NOP53 regulates autophagy both through the *ZKSCAN3*−dependent and −independent pathways. (**A**) NOP53 expression was upregulated in the control cells or *ZKSCAN3* knockdown cells by transfecting siRNA targeted to *ZKSCAN3* (siZK3) with or without the rapamycin treatment for 12 h. Then, the cells were immunocytochemically stained using an anti−LC3B antibody. The histogram shows the ratio of LC3B−positive cells. Data were obtained from three independent experiments and shown as the means ± SD; * *p* < 0.01. (**B**) Cells were transduced with adenovirus−expressing GFP (Ad−GFP), *ZKSCAN3* (Ad−ZK3), or NOP53 (Ad−NOP53) for 24 h, and then, a qPCR (left panel) and Western blots (right panel) were carried out. The histogram data obtained from three independent experiments represent a fold increase in the mRNA level of each target gene relative to the basal level of the control cells. * *p* < 0.01. (**C**) mRNAs were extracted from siSCR, siZK3, and siNOP53 cells, and a qPCR was performed as in “B”. * *p* < 0.01. (**D**) Cells were transfected with siZK3 for 48 h, followed by an Ad−GFP or Ad−NOP53 infection for an additional 24 h, and a qPCR (left panel) was performed. The histogram data obtained from three independent experiments represent a fold increase in the mRNA level of each target gene relative to the basal level of the control cells. * *p* < 0.01. Representative immunoblot images are shown in the right panel.

To investigate the role of *ZKSCAN3* in autophagy flux through NOP53, we determined whether *ZKSCAN3* knockdown restores the autophagy flux suppressed by NOP53. *ZKSCAN3* knockdown LN18 cells or control cells with *ZKSCAN3*-targeted small interfering RNA (siZK3) or scrambled siRNA (siSCR) transfection, respectively, were transduced with Ad-NOP53, and autophagy was measured by counting the cells with LC3B spot formations after the rapamycin treatment. As shown in Figure 4A, the autophagy-suppressing effect of NOP53 was reduced by the *ZKSCAN3* knockdown, suggesting that *ZKSCAN3* mediates the effects of NOP53 on autophagy. Interestingly, however, a *ZKSCAN3* knockdown could not restore rapamycin-induced autophagy to the level of the control cells (Figure 4A), suggesting that autophagy suppression by NOP53 was achieved through both the *ZKSCAN3*-dependent and -independent pathways. In order to clarify these points, we determined the changes of the expression levels of the ATGs after NOP53 or *ZKSCAN3* overexpression. Real-time qPCR assays and Western blots revealed that NOP53 downregulated the expressions of *ATG7*, *ATG12*, and LC3B while *ZKSCAN3* suppressed LC3B expression (Figure 4B). In addition, we found an upregulation of *ATG7* and *ATG12* in NOP53-knockdowned cells by siNOP53 transfection (siNOP53) and LC3B both in siNOP53 and siZK3 cells (Figure 4C). However, the expression levels of *ATG7* and *ATG12* were not affected by the *ZKSCAN3* expression level (Figure 4B,C). To support the *ZKSCAN3*-independent regulation of *ATG7* and *ATG12*, we knocked down *ZKSCAN3* with a siRNA transfection, followed by transducing with Ad-GFP or Ad-NOP53. As depicted in Figure 4D, NOP53 suppressed the expression of *ATG7* and *ATG12*, irrespective of *ZKSCAN3*. Taken together, these results indicate that NOP53 suppresses autophagy by downregulating a set of ATGs through both the ZKSCAN-dependent and -independent pathways.

### 2.5. NOP53 Interacts with Histones and Suppresses Phosphorylation of Histone H3 at S10

**Figure 5:**
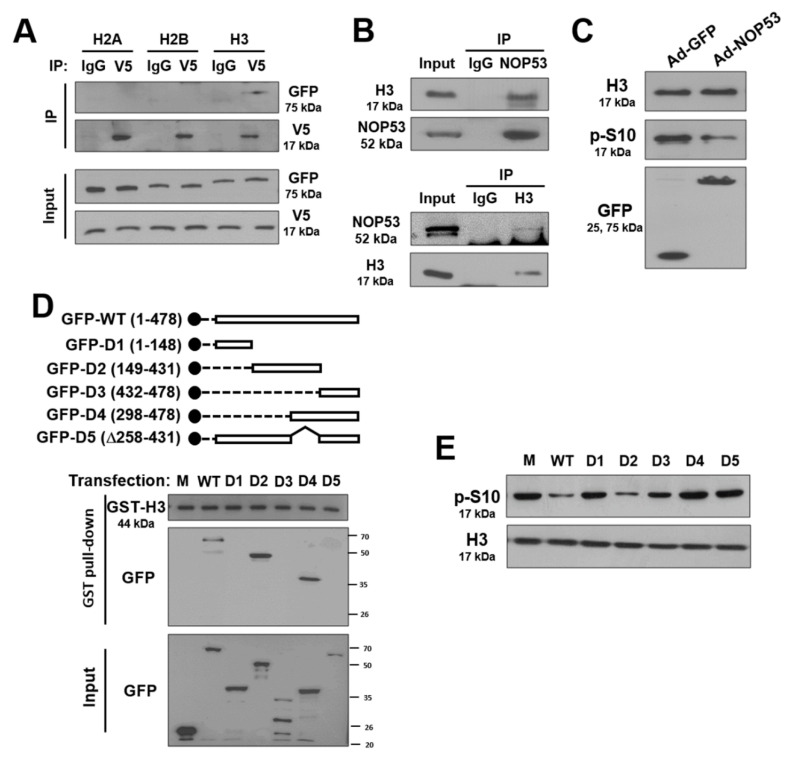
NOP53 interacts with histone H3. (**A**) Cells were co-transfected with V5-tagged NOP53-expressing plasmid and each set of GFP-tagged histone-expressing plasmids, as indicated (H2A, H2B, H3). Cell lysates were immunoprecipitated (IP) with an anti-V5 (V5) or isotype-matched control antibody (IgG). The immune precipitates were subjected to Western blotting using the indicated antibody (upper panel). Western blot images from whole-cell lysates (input) are shown in the lower panel. (**B**) Endogenous NOP53 and histone H3 interact with each other. The cell lysates from LN18 cells were immunoprecipitated with an anti-NOP53 (upper panel) or anti-H3 antibody, and Western blotting was conducted with the indicated antibody. (**C**) The cells were transduced with Ad-GFP or Ad-NOP53, and Western blotting was carried out with anti-H3, anti-phosphoserine, and anti-GFP antibodies. (**D**) Mock (M), GFP-tagged wild-type NOP53 (WT), and a series of mutant NOP53 (D1, D2, D3, D4, &D5) were generated (upper panel). The cells were transfected with indicated plasmid constructs and the cell lysates were mixed with GST-H3 protein bound to glutathion-sepharose beads for 1 h. GST pulldown was performed and Western blotting was carried out with indicated antibodies (lower panel). (**E**) Mock, NOP53, and a set of NOP53 mutant plasmids were ectopically expressed in LN18 cells, and Western blotting was performed with the indicated antibody.

Covalent modifications of histones are one of the key epigenetic mechanisms for gene expression [25]. Thus, we examined whether NOP53 affected the histone modification in the process of autophagy regulation. Initially, we determined whether NOP53 interacts with histones. The cells were co-transduced with V5-tagged NOP53-expressing plasmid (NOP53/V5) and histone-expressing plasmid tagged with GFP (pGFP-H2A, -H2B, or -H3), and the association of NOP53 with histones was determined by immunoprecipitation. Ectopic NOP53 was specifically precipitated with histone H3 but not with H2A or H2B (Figure 5A). The interaction between endogenous NOP53 and histone H3 is shown in Figure 5B. Next, to elucidate the effects of a NOP53–Histone H3 interaction on histone modification, we performed a Western blot using antibodies to detect the phosphorylation, acetylation, and methylation of histone H3. Interestingly, we found that the phosphorylation of histone H3 at S10 was suppressed by NOP53 (Figure 5C). To determine whether the interaction between NOP53 and histone H3 effected the phosphorylation on S10, we mapped NOP53 and found that the amino acids 258–431 of NOP53 are crucial for histone H3 binding (Figure 5D). In addition, transduction with a set of plasmid constructs, including D5 lacking the histone H3-binding domain (D5), revealed that the NOP53–histone H3 interaction is important for dephosphorylation at S10 (Figure 5E). Taken together, our findings indicate that NOP53 suppresses the phosphorylation of histone H3 at S10 by a direct interaction.

### 2.6. Histone H3 Dephosphorylation at S10 Is Crucial for ATG Suppression by NOP53

**Figure 6:**
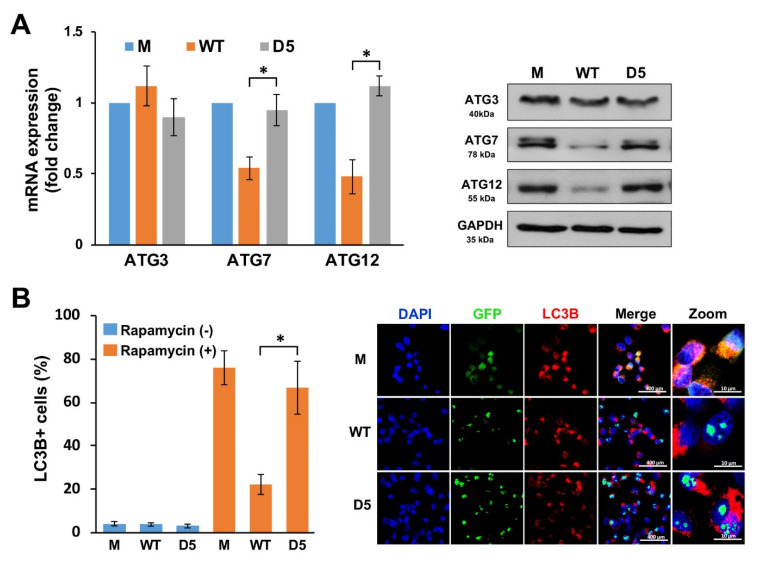
NOP53 reduces *ATG7* and *ATG12* expressions through interactions with histone H3. (**A**) Mock (M), wild−type NOP53 (WT), and the D5 mutant (D5) were transfected in LN18 cells for 48 h, and then, qPCR (left panel) and Western blotting (right panel) were conducted. Data were obtained from three independent experiments and shown as the means ± SD; * *p* < 0.01. (**B**) The cells were treated as in “A” with or without rapamycin, and immunocytochemical staining was performed using an anti−LC3B antibody. The histogram reveals the ratio of cells with LC3B−positive cells. Data were obtained from three independent experiments and shown as the means ± SD; * *p* < 0.01. Representative immunocytochemical images are shown in the right panel.

To investigate the effects of NOP53–histone interactions on the expression of ATGs, we transduced cells with the wild-type or D5-mutant plasmids of NOP53, and the expression levels of *ATG7* and *ATG12* were determined by qPCR and Western blotting. As shown in Figure 6A, the expression of D5 failed to suppress both the mRNA and protein levels of *ATG7* and *ATG12*. Consistent with these observations, D5 was not able to suppress the autophagy induction by rapamycin (Figure 6B). Together, our results demonstrate that the dephosphorylation at S10 of histone H3 by a NOP53–histone H3 interaction is crucial for the *ZKSCAN3*-independent autophagy regulation of NOP53.

## 3. Discussion

The current autophagy studies are often confused, because they involve apparently contradictory roles, such as survival and cell death, depending on the model used [26]. Autophagy plays a role in cell survival during nutrient deprivation or exposure to hypoxia, whereas autophagy can serve as a potent death signal in response to certain cellular stresses. Similarly, our study contradicts a previous investigation, which suggested that NOP53 overexpression promotes autophagic cell death, resulting in the inhibition of growth and proliferation of cells [27]. NOP53 inhibited upstream binding factor (UBF) phosphorylation, which is required for rRNA polymerase I activation during rRNA transcription and inhibited the AKT/mTOR/p70S6K pathway that regulates autophagy. On the other hand, our study showed that NOP53 overexpression inhibits autophagosome formation and NOP53 knockdown augmented autophagy. NOP53 transcriptionally regulates the expression of *ZKSCAN3*, which subsequently downregulates autophagosome formation. In this study, to minimize the cellular stress caused by plasmid transfection, we utilized an adenovirus with the Tet/OFF system instead of plasmid transfection for NOP53 overexpression. We also checked the endogenous LC3B instead of exogenous LC3B protein. Therefore, the basal level of autophagosome formation (endogenous LC3B-II level) was low in the non-stimulated conditions, even though NOP53 was overexpressed. However, the autophagy induction conditions showed the inhibition of autophagy formation by NOP53 overexpression. Depending on the physiologic or environmental conditions, such as nutrient deprivation or cellular stresses, as well as the method assessing the autophagy levels in each cell, a wide variety of autophagic responses might be shown, providing a plausible reason for why the results of these two investigations contradict each other.

ATG proteins play a pivotal role in the autophagy pathway. For autophagosome formation, the *ATG12*-*ATG5* and ATG8/LC3-phosphatidylethanolamine (LC3-PE) conjugation processes are essential [28]. To generate LC3-PE for autophagosome formation, three conjugates are sequentially generated. First, LC3-I is conjugated with and activated by *ATG7*, an E1-like enzyme. The LC3-*ATG7* is exchanged with *ATG3*, an E2-like enzyme, forming a LC3-*ATG3* conjugate. Finally, LC3-*ATG3* is exchanged with PE to form LC3-II by the E3-like enzyme *ATG16L1*/*ATG5*-*ATG12* complex [29]. The inhibition of Atg 7 reduces the autophagic response [30]. Moreover, autophagy is indirectly regulated by histone H3 modification. For example, histone H3 hyperacetylation, resulting from an acetyl-coenzyme A synthetase 2 (ACS2)-mediated increase in nucleocytosolic acetyl-coenzyme A (AcCoA) biosynthesis, participates in the transcriptional reduction of several autophagy-essential ATG genes (*ATG5*, *ATG7*, and ATG14) [31]. In addition, the phosphorylation of S10 in histone H3 is linked to the transcriptional activation of a specific subset of the histone deacetylase (HDAC) family, and class IIb HDACs regulate *ATG7* and LC3 [32]. In this study, NOP53 reduced the *ATG7* and *ATG12* levels under the condition of autophagy induction and decreased the histone H3 S10 phosphorylation level. According to these results, NOP53 inhibited autophagy formation by reducing *ATG7* and *ATG12*, which could be mediated by histone H3S10 dephosphorylation. However, further studying is required to produce definite proof of the link to the NOP53–H3 S10 phosphorylation–ATG gene axis.

*ZKSCAN3* is a master transcriptional repressor of autophagy [24]. *ZKSCAN3* transcriptionally modulates the expression of various genes involved in autophagy processes and lysosome biogenesis/functions. *Map11c3b* encoding cytosolic LC3B plays an important role in the formation and maturation of autophagosomes and is present in autophagosomes throughout the maturation process [24]. *Stx5*, *Ubqln2*, *Sec22b*, and *Bet1* are involved in autophagosome–lysosome fusion [33,34]. *Ulk1*, *Wipi2*, and *Dfcp1* play a role in the initiation of autophagosome biogenesis [35,36,37]. *Dira3*, *Bad*, *Sapk1*, and *RelA* are involved in the modulation of autophagy regulation. Herein, we show that NOP53 transcriptionally regulates the expression of *ZKSCAN3* and suppresses the autophagy process. However, the NOP53-mediated transcriptional regulation of *ZKSCAN3* is indirect and remains to be elucidated.

In this study, we show a novel nucleolar–cytoplasmic axis regulating the cytoplasmic autophagy process. NOP53 regulates the autophagic flux through divergent pathways: *ZKSCAN3*-dependent and -independent. In the *ZKSCAN3*-dependent pathway, NOP53 transcriptionally activates the master autophagy suppressor *ZKSCAN3*, thereby inhibiting LC3B induction and autophagy propagation. In the *ZKSCAN3*-independent pathway, NOP53 physically interacts with histone H3 to dephosphorylate the S10 of H3, which, in turn, transcriptionally downregulates the expression of *ATG7* and *ATG12*. Our results identify nucleolar protein NOP53 as a member of the nucleolar-nuclear–cytoplasmic axis in autophagy regulation.

## 4. Materials and Methods

### 4.1. Cell Culture, Antibodies, and Reagents

The LN18 and HEK293T cells were obtained from the Korean Cell Line Bank (Seoul, Korea) and cultured in Dulbecco’s modified Eagle’s medium (DMEM) supplemented with 10% fetal bovine serum. The anti-NOP53 rabbit polyclonal antibody was purified as previously described [16]. The anti-LC3B, anti-H3, anti-phospho-H3, anti-*ATG12*, anti-*ATG7*, and anti-*ATG3* antibodies were purchased from Cell Signaling Technology (Danvers, MA, USA). The anti-*ZKSCAN3* and anti-*GAPDH* antibodies were obtained from Santa Cruz Biotechnology (Santa Cruz, CA, USA). Unless otherwise specified, all other reagents were purchased from Sigma-Aldrich (St. Louis, MO, USA).

### 4.2. Plasmid and Viral Constructions and Transduction

Plasmids expressing GFP-tagged H2A, H2B, and H3; the GFP-tagged D1, D2, D3, D4, and D5 mutants of NOP53; and plasmids expressing p53 or NPM were generated through standard cloning techniques or splicing overlap extension, as described previously [16]. The cells were transfected with plasmids using Lipofectamine 3000 (Thermo Fisher Scientific Inc., Waltham, MA, USA) according to the manufacturer’s protocols. The construction of adenovirus-expressing GFP-tagged NOP53 has been previously described [38]. The knockdown of NOP53 was performed by a lentivirus construction using shNOP53 (Santa Cruz) and the establishment of a stable cell line through puromycin selection. The knockdown of *ZKSCAN3* was carried out by the transient transfection of siRNA targeted at *ZKSCAN3* (Santa Cruz) using Lipofectamine RNAiMAX. Briefly, the cells were plated in 24 wells one day prior to transfection. Two microliters of 20 µM siRNA in Opti-MEM I (Thermo Fisher Scientific Inc., Waltham, MA, USA) was mixed with 5 µL of RNAiMAX in Opti-MEM I and incubated for 20 min at room temperature. After replacing the medium in the plate, a mixture of siRNA and RNAiMAX was added to the cells. After 12 h, the medium was replaced with DMEM, and the cells were cultured for 72 h before the analysis was performed.

### 4.3. Real-Time qPCR and Promoter Assays

mRNA was isolated with the Qiazol Lysis Reagent (Qiagen, Germantown, MD, USA). Total RNA (1 mg) was reverse-transcribed using Moloney Murine Leukemia Virus Reverse Transcriptase (Gibco BRL, Waltham, MA, USA) with random hexamer priming. To determine the expressions of the genes, a real-time PCR analysis was performed with the LightCycler-FastStart DNA Master SYBR Green I mix (Roche Diagnostics, Basel, Switzerland) using specific primers for each target gene and glyceraldehyde-3-phosphate dehydrogenase (Table 1). The fold changes were calculated using the 2^−ΔΔCt^ method.

The *ZKSCAN3* and *CHOP* promoter sequences were amplified by PCR from human kidney genomic DNA using upstream and downstream primers (Table 1). Each promoter fragment was cloned in-frame into a pGL luciferase reporter vector to generate pGL-*ZKSCAN3* and pGL-*CHOP*. The luciferase activity was measured in the samples containing equivalent amounts of proteins using the Dual-Luciferase Reporter Assay System (Promega, Madison, WI, USA).

### 4.4. Immunofluorescence Microscopy and Quantitative Analysis

For immunocytochemical staining, the cells were fixed with methanol for 7 min, blocked with 1% BSA for 30 min, and incubated with the indicated antibodies at 4 °C for 16 h, followed by incubation with a secondary antibody at room temperature for 1 h. After nuclear staining with DAPI, the cells were observed using an inverted confocal microscope (LSM 510 META; Zeiss, Jena, Germany). For the quantification, 150 cells were counted in three independent experiments for each group. The cells containing 5 or more LC3B puncta were scored as LC3B+ cells.

### 4.5. Western Blotting and Immunoprecipitation

Western blotting was carried out as described previously [38]. For Western blotting, the anti-NOP53 (1:100), anti-LC3B (1:200), anti-H3 (1:1000) and anti-S10 H3 (1:1000), anti-*ATG3* (1:100), anti-*ATG7* (1:100), anti-*ATG12* (1:100) and anti-*ZKSCAN3* (1:100), and anti-*GAPDH* (1:2000) antibodies were used. For the immunoprecipitation, the cells were lysed using a lysis buffer (50-mM Tris-HCl, pH 7.5, 500-mM NaCl, 0.5% Triton X-100, 1-mM EDTA, and 1-µM DTT). After centrifugation, the lysates were incubated with the indicated antibodies (5 μg) for 16 h and immunoprecipitated with 20 μL of protein A (GE Healthcare, Chicago, IL, USA) for 1 h. The precipitate was washed 4 times with an RIPA buffer, and Western blotting was performed.

### 4.6. Recombinant Proteins and Pull-Down Assays

The recombinant H3-GST fusion protein was produced by transfecting pGEX4T-H3 to *Escherichia. Coli* and IPTG induction and purified using a column filled with glutathione resin (Thermo Fisher Scientific Inc., Waltham, MA, USA). The recombinant NOP53-GST protein was purchased from Abnova Corporation (Taipei, Taiwan).

One hundred nanograms of the GST fusion protein were bound to glutathione-sepharose beads in phosphate-buffered saline (PBS) at 4 °C for 30 min. After washing with RIPA buffer, the beads were mixed with lysates of HEK 293T cells transfected with the indicated plasmids. The samples were centrifuged and washed with the RIPA buffer four times. The bound proteins were dissociated by boiling, and then, the results were confirmed by Western blotting.

### 4.7. Statistical Analysis

The statistical analysis was carried out using SPSS software, version 13.0 (SPSS, Armonk, NY, USA). Data were analyzed with the Student’s *t*-test. Differences with a *p*-value less than 0.05 were considered statistically significant.

## Figures and Tables

**Table 1:** The primer sequences used for real-time quantitative PCR or promoter cloning.

Gene	Forward Primer	Reverse Primer
*ATF4*	CTTACGTTGCCATGATCCCT	TCCCATCTCCAGGTGTTCTC
*CHOP*	TGGAAGCCTGGTATGAGGAC	CAGAACCAGCAGAGGTCACA
*FOXO1*	TGGACATGCTCAGCAGACATC	TGAACCGCCTGACCCAA
*FOXO3*	AGAAGTTCCCCAGCGACTTG	GTTGGTTTGAACGTGGGGA
*SREBF2*	AGGAGAACATGGTGCTGA	TTGACTCTGAGCCAGGAA
*STAT1*	CGGTTGAACCCTACACGAAG	ACCAGAGCCAATGGAACTTG
*STAT3*	AGCAGCACCTTCAGGATGTC	AGTGACCAGGCAGAAGATGC
*ZKSCAN3*	GGTCTCCCTGGGTGATGAAA	GCACATGTAGGAATCTGGGC
*MAPILC3B*	ACGATACAAGGGTGAGAAGCA	GACCATGCTGTGTCCGTTC
*ATG3*	GTTGGAAACAGATGAGGCTACC	TAGCCAAACAACCATAATCGTG
*ATG4A*	GTGCTCGTCTATGGTTTACATAC	AATACCAACGCATCCTACAGTGC
*ATG5*	ACCAGTTTTGGGCCATCAAT	GTGTGTGCAACTGTCCATCTG
*ATG7*	AAGCAAGAGAAAGCTGGTCATC	AGTAGCAGCCAAGCTTGTAACC
*ATG9A*	CTCATGCAGTTCCTCTTTGTGGT	GTGCCAGGATTCAGGAAAATGG
*ATG10*	CTGAAGGACATATGGGAAGGAG	GAGGTAGATTCAGCCCAACAAC
*ATG12*	GCAGCTTCCTACTTCAATTGCT	ATTGCAGAATGTTTGCAGACTA
*ATG16L1*	AAATGGCCCAACTGAGGATTAA	ATTGCAGAATGTTTGCAGACTA
*GAPDH*	GGCATGGACTGTGGTCATGAG	GGCATGGACTGTGGTCATGAG

## Data Availability

Not applicable.
